# Incidence rate of alcohol use disorder and correlation between metabolic changes and addictive behavior in patients after sleeve gastrectomy

**DOI:** 10.3389/fpsyt.2025.1652020

**Published:** 2025-10-21

**Authors:** Fuqin Wang, Youjie Fan, Jianhua Li, Jingwang Yan, Zhiyong Li

**Affiliations:** ^1^ Department of General Surgery, Xinxiang Central Hospital, Xinxiang, Henan, China; ^2^ Department of Psychiatry, Xinxiang Central Hospital, Xinxiang, Henan, China

**Keywords:** sleeve gastrectomy, alcohol use disorder, metabolic changes, addictive behavior, correlation analysis

## Abstract

**Aim:**

To compare the incidence of AUD and correlation between metabolic changes and addictive behaviors in patients who underwent SG.

**Methods:**

A retrospective study was conducted on 160 obese patients who underwent SG treatment at our hospital between February 2023 and April 2024 (SG group), and another 160 non-surgical obese patients admitted during the same period were selected as the control group. The Alcohol Use Disorders Identification Test (AUDIT)was used to assess the risk of AUD in both groups (AUDIT≥eight points defined as high risk) and to compare the differences in high-risk rates between the groups. Differences in impulsivity scores (Barratt Impulsivity Scale [BIS]-11), addictive behavior scores(Visual Analog Scale for Addictive Behaviors[VAS]),and glucose-fat metabolism indexes between the high-and low-risk AUD subgroups within the SG group were analyzed using Pearson correlation and multiple regression analyses to explore associations between metabolic indicators and addictive behavior scores.

**Results:**

The SG group had a higher rate of alcohol use disorder (AUDIT ≥ 8 points) after surgery than the control group (26.88% vs. 8.125%) (χ² = 19.32, P < 0.001).The impulsivity score[BIS-11:(68.43 ± 9.35)points vs.(61.22 ± 8.71)points] and addictive behavior score[VAS:(6.42 ± 1.14)points vs.(3.88 ± 1.06)points]were significantly higher in the high-risk group than in the low-risk group(P<0.001).Fasting plasma glucose, glycated hemoglobin, and homeostasis model assessment of insulin resistance levels were significantly higher in the AUDIT high-risk group than in the low-risk group(P<0.001).lipoprotein cholesterol (TC, TG, LDL-C, HDL-C) did not differ significantly between high- and low-risk groups (P > 0.05).Glucose metabolism indices(fasting plasma glucose, glycated hemoglobin, and homeostasis model assessment of insulin resistance)were strongly and positively correlated with AUDIT and VAS scores(r=0.682–0.716,P<0.05).However, multivariate linear regression analysis indicated that impulsivity, addictive behavior propensity, and glucose metabolism abnormalities were not independently associated with statistical significance(P>*0*.05).The propensity for addictive behavior and abnormal glucose metabolism remained independent risk factors for AUD after SG(P<0.05),and the risk was significantly higher in men than in women. This age group had significantly higher AUDIT high-risk rates, BIS-11 impulsivity, and VAS addiction behavior scores vs. the >25 group (P<0.05).

**Conclusion:**

Compared to nonsurgical patients with obesity, patients with obesity who underwent SG exhibited a significantly high incidence of AUD. Patients in the high-risk subgroup for AUD also showed high impulsivity scores, greater addictive behavior scores, and notable abnormalities in glucose metabolism indices.

## Introduction

As obesity rates continue to increase globally, metabolic weight-loss surgery—particularly sleeve gastrectomy (SG)—is widely used to treat severe obesity and its associated metabolic disorders (1). This procedure significantly reduces gastric volume by removing approximately 80% of the gastric body from the greater curvature of the stomach and affects the secretion of gastrointestinal hormones, thereby improving metabolic status and reducing body weight (2). Despite the significant advantages of SG in improving type 2 diabetes mellitus, hypertension, and sleep apnea, long-term behavioral and psychological changes in patients after surgery, especially the transfer of addictive behaviors, have raised clinical concerns. In recent years, studies have noted (3, 4) that some patients experience an increased risk of developing alcohol use disorder (AUD) following SG. This phenomenon may be related to accelerated gastric emptying, changes in blood–brain barrier permeability, altered neurotransmitter sensitivity in the reward system, and postoperative fluctuations in hormone levels. Decreased satisfaction with food-related pleasure in some patients after surgery may prompt them to seek alternative forms of stimulation, such as alcohol, to satisfy the reward mechanisms of the dopamine system (5). In addition, patients after SG experience significant metabolic changes, including in glucose, lipid, and amino acid metabolism, which are closely linked to central nervous system function; alterations in these metabolites can affect emotional regulation, self-control, and susceptibility to addiction (6). However, few studies have examined the incidence of AUD, metabolic changes, and addictive behaviors in this population. Based on these considerations, the present study was conducted to compare the incidence of AUD between patients who underwent SG and nonsurgical patients with obesity, analyze the association between metabolic indicators and impulsivity and addictive behaviors within the SG group, and provide a scientific basis for behavioral interventions, presurgical risk assessment, and long-term follow-up management of patients after SG.

## Materials and methods

### General information

The sample size was calculated based on a comparison of the rates between the two groups using the following formula:


$$n={\left(\frac{Z_{\alpha /2}+Z_{\beta }}{P_{1}-P_{2}}\right)}^{2}\times \left(\frac{P_{1}\left(1-P_{1}\right)+P_{2}\left(1-P_{2}\right)}{{\left(\Delta \right)}^{2}}\right)$$

Where: Z_α/2_ is the normally distributed value when the significance level is α/2 (two-sided test); Z_β_ is the normally distributed value when the test efficacy is 1-β; p_1_ and p_2_ are the expected values of the high-risk rate of AUD for the SG and control groups, respectively; and Δ is the minimum clinically significant difference between the two groups. Based on the data from previous studies and pretests, the sample size required for each group was calculated to be approximately 150 cases, assuming a high-risk rate of AUD of 25% (3)in the SG group and 10% (4) in the control group, taking α = 0.05 (two-sided) and β = 0.20 (80% test efficacy). Considering possible non-response and data loss, and ensuring balanced sample sizes between the two groups, the final decision was made to include 160 cases in the SG group and 160 cases in the control group.

This study included 160 obese patients who underwent SG as the study subjects (SG group) and selected 160 non-surgical obese patients treated during the same period as the control group and the cases were selected from February 2023 to April 2024. In the SG group, there were 75 men and 85 women, with ages ranging from 18 to 64 years, and the preoperative body mass index (BMI) ranged from 31.57 to 40.53 kg/m². In the control group, there were 77 men and 83 women, aged 17 to 65 years, with preoperative BMI of 31.63 to 40.49 kg/m². General information on the two groups is shown in **Table 1**, which was statistically analyzed and showed no statistical difference (P > 0.05). This study was approved by our institutional ethics committee.

**Table 1:** Comparison of general information and high-risk rate of AUDIT between the two groups (n/%, $\bar{\text{x}}$ *± s*).

Item	SG Group (n=160)	Control group (n=160)	*t/χ^2^*	*P*
Sex (male/female)	75/85	77/83	0.031	0.860
Age (years)	42.78± 6.81	42.95 ± 7.10	-0.198	0.843
Preoperative BMI (kg/m^2^)	37.56± 2.18	37.40 ± 2.35	0.624	0.533
FPG (mmol/L)	6.82± 1.35	6.78 ± 1.43	0.249	0.804
HbA1c (%)	6.58± 0.92	6.53 ± 1.54	0.331	0.741
HOMA-IR	4.86± 1.72	4.89 ± 1.63	-0.160	0.873
TC (mmol/L)	5.21± 0.89	5.20 ± 0.92	0.099	0.921
TG (mmol/L)	2.37± 0.68	2.39 ± 0.70	-0.254	0.800
LDL-C (mmol/L)	3.42± 0.75	3.40 ± 0.78	0.230	0.818
HDL-C (mmol/L)	0.98± 0.21	1.00 ± 0.20	-0.888	0.375
AUDIT high-risk rate (n/%)	43 (26.88)	13(8.13%)	19.320	<0.001
BIS-11 score (points)	62.35 ± 8.42	61.90 ± 8.59	0.467	0.641
VAS score (points)	3.95 ± 1.83	3.85 ± 1.15	0.581	0.562
AUDIT score (points)	4.27 ± 1.83	4.08 ± 1.81	0.953	0.341

AUDIT, Alcohol Use Disorders Identification Test; BIS-11, Barratt Impulsiveness Scale; VAS, Visual Analogue Scale for Addictive Behaviors; BMI, Body Mass Index; FPG, Fasting Plasma Glucose; HbA1c, Glycated Hemoglobin; HOMA-IR, Homeostatic Model Assessment of Insulin Resistance; TG, Triglycerides; TC, Total Cholesterol; Low-Density Lipoprotein Cholesterol (LDL-C); HDL-C, High-Density Lipoprotein Cholesterol.

Inclusion criteria: (1) age 18 to 65 years, meeting the diagnostic criteria for severe obesity (7) (BMI ≥ 35 kg/m² or BMI ≥ 30 kg/m² combined with at least one obesity-related metabolic disease, such as type 2 diabetes mellitus and hypertension); (2) first-time treatment with SG, with no history of other bariatric surgeries; (3) no preoperative history of AUD (Alcohol Use Disorder Identification Test [AUDIT] < eight points) or history of substance abuse; (4) full postoperative follow-up for more than 6 months with good compliance; (5) the control group met the criteria for severe obesity but did not undergo any bariatric surgery and had no history of alcohol dependence.

Exclusion criteria were as follows: (1) patients with combined serious organic diseases; (2) previous history of mental illness or long-term use of psychotropic drugs; (3) alcohol dependence or other addictive behaviors already present before the operation; and (4) pregnant or lactating women.

## Methods

### Psychological and behavioral assessment

Demographic information (age and sex), BMI, and scores on psycho-behavioral scales [AUDIT (8), Barratt Impulsivity Scale (BIS-11) (9), and Visual Analog Scale for Addictive Behaviors (VAS) (10)] were collected through the hospital’s internal medical record system. The AUDIT scale contains 10 items, including three aspects: frequency of drinking, amount of drinking, symptoms of dependence, and drinking-related harm. Each entry is scored from zero to four points according to its severity (including “number of drinks per month”: zero = never, four = daily or almost daily), and the total score ranges from zero to 40 points. According to the international consensus: low risk: 0–7 points (no or mild drinking problem); high risk (AUD tendency): ≥ eight points. (ii) The BIS-11 contains 30 items divided into three dimensions: attentional impulsivity (cognitive), motor impulsivity (behavioral), and unplanned impulsivity (decision-making). A four-point Likert scale (one = “never” to four = “always”) was used, with a total score range of 30 to 120. High total scores indicate poor impulse control. (iii) The VAS quantifies the intensity of subjective craving by a self-report method using a 10 cm straight line (zero to 10 points) anchored at both ends as zero: no craving at all (including “don’t want to drink at all”) and 10: extremely strong craving (including “strongest desire to drink in life”). Participants marked the location of their current craving for alcohol, with a score of ≥ 4 indicating a significant tendency toward addictive behavior.Scale assessments were standardized to be conducted 6 months postoperatively.

### Metabolic index testing

Five milliliters of elbow venous blood were collected from patients in the SG group at 6 months postoperatively, and the serum was separated by centrifugation (3000 rpm for 10 min) and tested. Glycated hemoglobin (HbA1c) levels were measured using a Bio-Rad Variant II Turbo Glycosylated Hemoglobin Analyzer. The test kit supported fasting blood glucose (FPG), total cholesterol (TC), triglycerides (TG), low-density lipoprotein cholesterol (LDL-C), and high-density lipoprotein cholesterol (HDL-C) levels. A Roche Cobas c 501 automatic biochemistry analyzer was used for determination, and the kits were used as matching test kits. All steps of the assay strictly followed the operating specifications of the kits and laboratory quality control procedures. The insulin resistance index was calculated as follows: Homeostatic Model Assessment of Insulin Resistance(HOMA-IR) = fasting insulin × FPG/22.5.

### Statistical methods

The data in this study were confirmed to be normally distributed by a normality test, and all statistical analyses were performed using SPSS 26.0. For categorical variables, the number of cases (n) and their corresponding percentages (%) were used for description, and the chi-square test (χ²) was utilized to compare different groups. Measured data that conformed to a normal distribution were expressed as ( $\bar{\text{x}}$ ± s), and an independent samples t-test was used to assess differences between groups. Pearson correlation was used to analyze the correlation between metabolic changes and addictive behaviors in patients who underwent SG. A multiple linear regression model was developed using the backward method in stepwise regression to explore potential influences on the risk of postoperative AUD in patients with SG. The goodness of fit of the model was evaluated by the adjusted R² value, and statistical significance was verified using ANOVA. P < 0.05 was considered statistically significant.

## Results

### General information and AUDIT high-risk rate across groups

The high-risk rate of postoperative AUD (AUDIT ≥ eight points) was 26.88% in the SG group and 8.13% in the control group (P < 0.001). There were no statistically significant differences (P > 0.05) in baseline data, including age, BMI, sex, or glycolipid metabolism indices, between the two groups (**Table 1**).

### Impulsivity and addictive behavior scores in AUDIT subgroups

**Figure 1:**
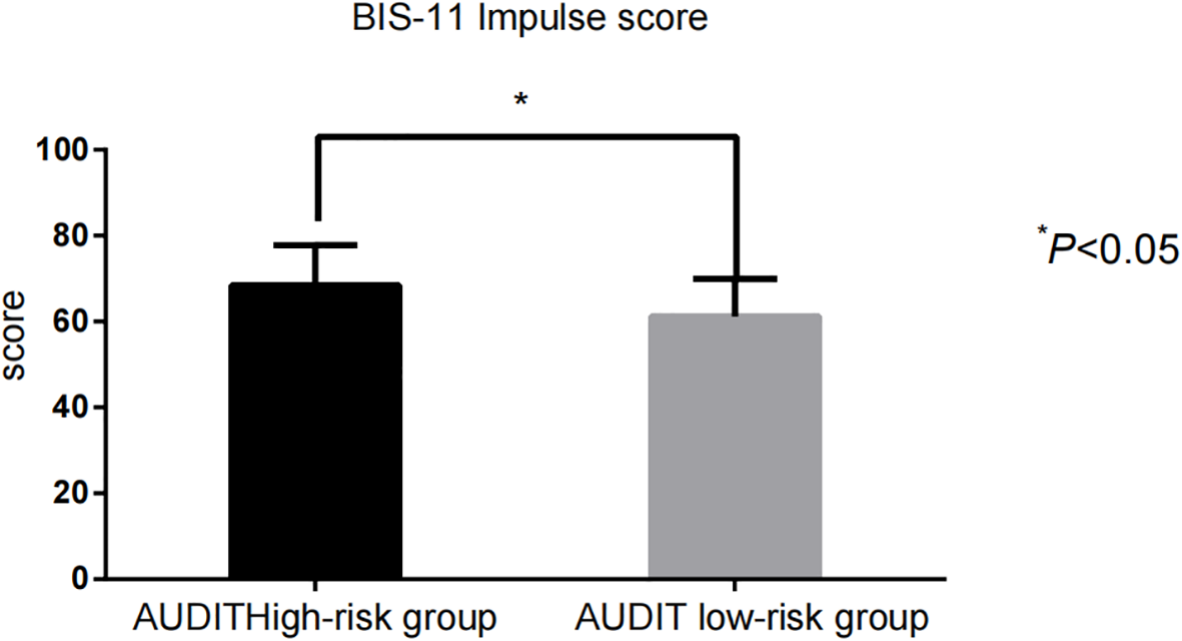
Comparison of BIS-11 impulsivity scores between AUDIT high- and low-risk groups.

**Figure 2:**
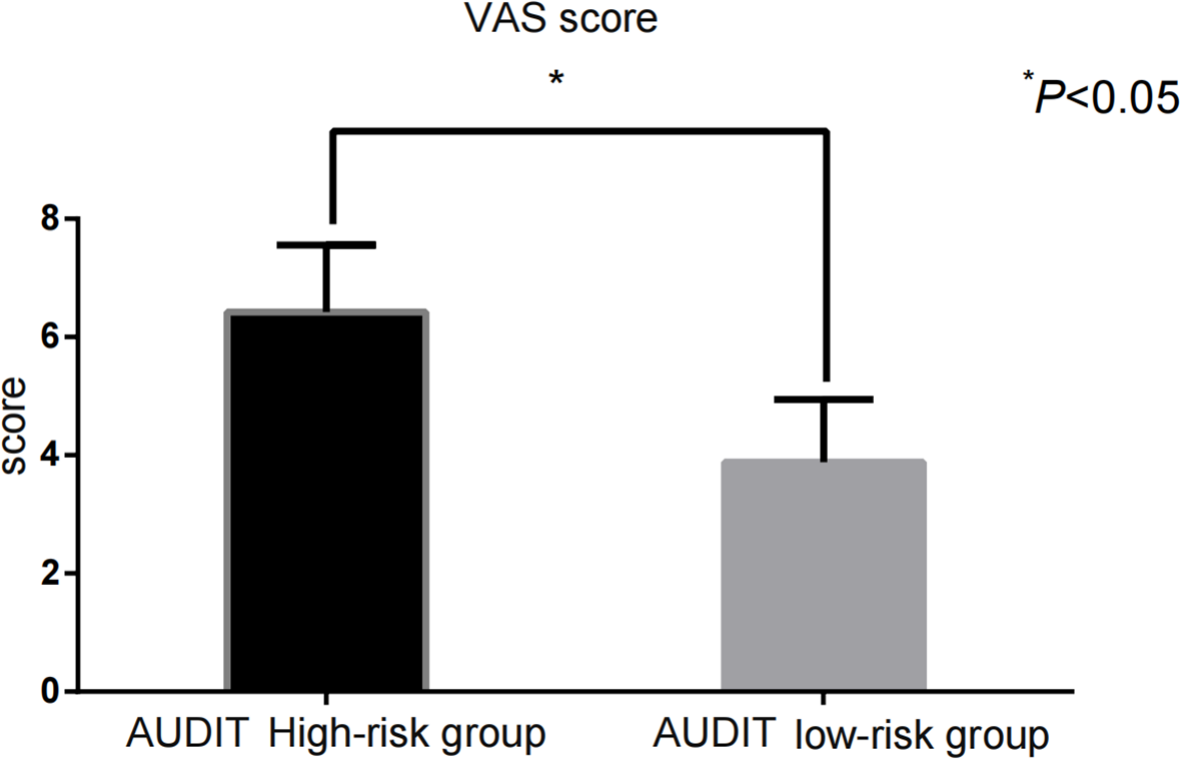
Comparison of VAS scores between AUDIT high and low-risk groups.

The impulsivity scores [BIS-11: (68.43 ± 9.35) vs. (61.22 ± 8.71)] and addictive behavior scores [VAS: (6.42 ± 1.14) vs. (3.88 ± 1.06)] in the AUDIT high-risk group were significantly higher than those in the low-risk group (P < 0.001) (**Figure 1**, **Figure 2**).

### Postoperative glucose metabolism in AUDIT subgroups

Postoperative levels of FPG, HbA1c, and Homeostatic Model Assessment for Insulin Resistance (HOMA-IR) were significantly higher in the AUDIT high-risk group than in the low-risk group (P < 0.001) (**Table 2**).

**Table 2:** Comparison of postoperative glucose metabolism indexes between AUDIT high-risk and low-risk groups ( $\bar{\text{x}}$ *± s*).

Subgroup	Cases	FPG (mmol/L)	HbA1c (%)	HOMA-IR
AUDIT high-risk group	43	4.31± 1.05	5.78± 0.82	2.95± 0.57
AUDIT low-risk group	117	3.46± 0.57	5.13± 0.35	1.06± 0.49
*t*		6.537	7.032	20.680
*P*		<0.001	<0.001	<0.001

AUDIT, Alcohol Use Disorders Identification Test; Fasting Plasma Glucose (FPG); Glycated Hemoglobin (HbA1c); Homeostatic Model Assessment of Insulin Resistance (HOMA-IR); independent samples t-test was used for intergroup comparisons.

### Postoperative lipid metabolism in AUDIT subgroups

There were no statistically significant differences in lipid metabolism indices—including TC, TG, LDL-C, and HDL-C—between patients in the AUDIT high-risk and low-risk groups (P > 0.05) (**Table 3**).

**Table 3:** Comparison of postoperative lipid metabolism indexes between AUDIT high-risk and low-risk groups ( $\bar{\text{x}}$ *± s*).

Grouping	Number of cases	TC (mmol/L)	TG (mmol/L)	LDL-C (mmol/L)	HDL-C (mmol/L)
AUDIT high-risk group	43	4.51± 0.73	1.32± 0.86	2.59± 0.53	1.15± 0.26
AUDIT low-risk group	117	4.33± 0.68	1.25± 0.79	2.61± 0.92	1.08± 0.23
*t*		1.455	0.485	0.134	1.647
*P*		0.148	0.628	0.893	0.102

AUDIT, Alcohol Use Disorders Identification Test; TG, Triglycerides; TC, Total Cholesterol; LDL-C, Low-Density Lipoprotein Cholesterol; HDL-C, High-Density Lipoprotein Cholesterol; independent samples t-test was used for intergroup comparisons.

### Correlations between metabolic indices, AUDIT, and VAS scores

Glucose metabolism indices (FPG, HbA1c, and HOMA-IR) were strongly and positively correlated with both the AUDIT and VAS scores (r = 0.682–0.716, P < 0.05). The BIS-11 impulsivity score was also significantly correlated with the AUDIT score (r = 0.669, P < 0.001) (**Table 4**).

**Table 4:** Results of correlation analysis of postoperative metabolic index changes with postoperative AUDIT score and VAS score.

Indicator	AUDIT score		VAS score	
	*r* value	*P* value	r-value	P-value
Glucose metabolism index				
FPG	0.716	<0.001	0.698	<0.001
HbA1c	0.682	<0.001	0.714	<0.001
HOMA-IR	0.704	<0.001	0.642	<0.001
Lipid metabolism index				
TC	0.124	0.054	0.126	0.081
TG	0.221	0.121	0.175	0.106 LDL-C
LDL-C	0.107	0.243	0.212	0.058
HDL-C	0.114	0.093	0.093	0.062
BIS-11 impulse score	0.669	<0.001	0.593	<0.001

AUDIT, Alcohol Use Disorders Identification Test; BIS-11, Barratt Impulsiveness Scale; VAS, Visual Analogue Scale for Addictive Behaviors; BMI, Body Mass Index; FPG, Fasting Plasma Glucose; HbA1c, Glycated Hemoglobin; HOMA-IR, Homeostatic Model Assessment of Insulin Resistance; TG, Triglycerides; TC, Total Cholesterol; LDL-C, Low-Density Lipoprotein Cholesterol; HDL-C, High-Density Lipoprotein Cholesterol.

### Multiple regression analysis of factors influencing the risk of AUD after SG

In this study, the presence or absence of AUD after surgery in patients who underwent SG was the dependent variable (yes = one, no = zero), and glucose metabolism indices (FPG, HbA1c, HOMA-IR), BIS-11 impulsivity scores, and VAS scores were included as continuous variables in the multiple linear regression model. Confounders included age (continuous variable) and sex (men vs. women). Impulsivity, propensity for addictive behaviors, and abnormal glucose metabolism remained independent risk factors for AUD after SG (P < 0.05), and the risk was significantly higher in men than in women (**Table 5**).

**Table 5:** Results of multiple regression analysis affecting the risk of AUD after SG surgery.

Variable	*β value*	*β’ value*	SE	*t-value*	*P-value*	*95%CI*
Constant term	-8.241	0.324	Constant term -8.241 0.324 -3.892	3.892	<0.001	-
BIS-11 score	0.512	0.412	0.336	6.732	<0.001	0.383~0.645
VAS score	1.863	0.228	0.512	3.812	<0.001	1.025~2.706
HOMA-IR	2.971	0.351	0.441	4.621	<0.001	1.854~4.097
FPG	1.822	0.228	0.654	3.105	0.003	0.756~2.896
HbA1c	1.765	0.201	0.108	2.987	<0.001	1.762~2.844
Sex	3.155	0.179	0.585	2.894	0.004	1.243~5.065
Age	-0.074	-0.042	0.241	0.912	0.363	-0.211~0.076

AUDIT, Alcohol Use Disorders Identification Test; BIS-11, Barratt Impulsiveness Scale; VAS, Visual Analogue Scale for Addictive Behaviors; BMI, Body Mass Index; Fasting FPG, Plasma Glucose; HbA1c, Glycated Hemoglobin; HOMA-IR, Homeostatic Model Assessment of Insulin Resistance; TG, Triglycerides; TC, Total Cholesterol; LDL-C, Low-Density Lipoprotein Cholesterol; HDL-C, High-Density Lipoprotein Cholesterol; The model was validated using analysis of variance (ANOVA): F = 43.821, P < 0.001; R² = 0.726, adjusted R² = 0.709.

### Subgroup analysis of SG patients aged 18–25 years

To further investigate the impact of age on the risk of AUD postoperatively, a subgroup analysis was conducted on patients aged 18–25 years in the SG group (n = 28, accounting for 17.5%). The results showed that the high-risk rate for AUDIT, BIS-11 impulsivity scores, and VAS addiction behavior scores were significantly higher in this age group than in the >25-year-old group (P<0.05) (**Table 6**).

**Table 6:** Comparison of AUD risk and related behavioral indicators between patients aged 16–25 and those aged >25 in the SG group.

Group	n	AUDI high-risk n (%)	BIS-11 score (points)	VAS score (points)
18~25years group	28	12	70.13 ± 8.94	6.87 ± 1.05
>25years group	132	31	61.75 ± 8.62	4.02 ± 1.12
t/χ2		χ²=4.411	t=4.643	t=12.35
P		0.036	<0.001	<0.001

AUDIT, Alcohol Use Disorders Identification Test; BIS-11, Barratt Impulsiveness Scale; VAS, Visual Analogue Scale for Addictive Behaviors; The AUD high-risk rate was compared using the chi-square test (χ²), and the BIS-11 and VAS scores were compared using the t-test.

## Discussion

In recent years, with the widespread use of metabolic weight-loss surgery, behavioral and psychological changes in postoperative patients have gradually received attention. SG, as a mainstream weight-loss surgical modality, is effective in reducing body weight and ameliorating metabolic disorders, while potentially triggering certain behavioral problems, such as AUD. Studies have shown (11–13) that the incidence of AUD is higher in patients after SG than in the general population, which may be related to the physiological and psychological changes caused by surgery. On one hand, the rapid weight loss and improved metabolic status of patients after SG may temporarily improve their self-esteem and quality of life, thereby increasing the risk of substance abuse, such as alcohol (14). On the other hand, the stress response during surgery and discomfort experienced during postoperative recovery may also motivate some patients to alleviate their anxiety and depression through substances, such as alcohol (15, 16). Therefore, alcohol use behavior in patients undergoing postoperative SG should be closely monitored, and timely interventions and guidance should be provided to prevent the occurrence of AUD.

In this study, the incidence of a high risk of postoperative AUD was significantly higher in patients who underwent SG than in obese controls who did not undergo surgery (P < 0.001), suggesting a trend toward an increased risk of addictive behaviors after SG. This finding is consistent with those of previous studies (17–19), suggesting that SG may increase the risk of postoperative AUD. This mechanism may be related to the following factors: physiological structural changes that accelerate alcohol absorption, gastrointestinal hormonal changes that interfere with the brain’s stress response and reward mechanisms, and changes in the neurotransmitter system that increase the craving for alcohol. Collectively, these factors are believed to contribute to an elevated risk of AUD in postoperative patients (20).

The mechanisms linking SG to an elevated AUD risk are multifactorial and extend beyond behavioral shifts. A critical factor is the alteration of alcohol pharmacokinetics. Post-surgery anatomical changes lead to faster gastric emptying and reduced first-pass metabolism, resulting in a quicker and higher peak in blood alcohol concentration (BAC) from the same amount of alcohol (21, 22). This heightened level of intoxication could reinforce drinking behavior. Furthermore, the interplay with gut hormones is complex. While metabolic surgery is known to increase endogenous levels of glucagon-like peptide-1 (GLP-1), a hormone associated with satiety, recent placebo-controlled studies show that acute alcohol consumption paradoxically decreases GLP-1 concentrations in both post-surgery and non-surgical individuals (21, 22). This reduction in a key satiation signal might contribute to alcohol’s “apéritif effect,” potentially undermining appetite control and promoting consumption. These hormonal and pharmacokinetic changes, combined with a heightened risk for alcohol-induced hypoglycemia observed in the post-surgical population (21, 22), create a complex physiological environment that could lower the threshold for developing AUD. It has also been shown that a higher Body Mass Index (BMI) is associated with a lower level of response to alcohol, a relationship largely accounted for by total body water, which influences alcohol concentration (23).

Regarding metabolic indices, this study found that glucose metabolic indices (FPG, HbA1c, and HOMA-IR) were significantly higher in the AUD high-risk group than in the low-risk group and were strongly and positively correlated with AUDIT and VAS scores (r = 0.682–0.716, P < 0.001), suggesting that abnormal glucose metabolism in the postoperative period may be closely related to addictive behaviors. This suggests that abnormal glucose metabolism may enhance sensitivity to alcohol-related reward stimuli by affecting brain insulin signaling, dopamine metabolism, and other pathways, which, in turn, increase the risk of addiction. In addition, abnormal insulin signaling in the nucleus ambiguous, a central neuromodulator, may be involved in the development of addictive behaviors (24, 25). By contrast, lipid metabolism indices (TC, TG, LDL-C, and HDL-C) did not differ significantly between the high- and low-risk groups and were not significantly correlated with AUDIT or VAS scores, suggesting that lipid metabolism does not have a significant effect on addictive behavior. This is because lipid metabolism primarily functions in energy storage and cellular structure maintenance, rather than influencing the central reward pathway (26).

The results of this study showed that the high-risk group for AUD had significantly higher BIS-11 impulsivity scores and VAS addiction behavior scores than the low-risk group. After adjusting for confounding factors such as age and gender, impulsivity, addiction behavior tendencies, and abnormal glucose metabolism remained independent risk factors for AUD risk after SG surgery (P<0.05), and male patients had a significantly higher risk than female patients (27). The risk was significantly higher in men than in women. This indicates that the presence of impulsivity, a tendency toward addictive behavior, abnormal glucose metabolism, and being a man not only correlate with a greater tendency to use alcohol but are also associated with more pronounced behavioral impulsivity and subjective craving. Individuals with high impulsivity lack self-control and are more likely to make impulsive drinking decisions when exposed to alcohol-related cues (25). Increased psychological stress in patients after SG, owing to various factors, such as altered body image, lifestyle adjustments, and metabolic changes, may further exacerbate impulsive tendencies, thereby increasing the risk of AUD (28). It is worth noting that alcohol consumption itself significantly impacts glycemic control by affecting hepatic glucose production, insulin secretion, and sensitivity. Therefore, the observed association between postoperative glycemic abnormalities and a high AUD risk may be bidirectional; drinking behavior could directly influence metabolic status, rather than the relationship being strictly causal in one direction. Although our correlation and regression analyses revealed a significant link, this study’s cross-sectional design cannot rule out this reverse causality. Future studies could better clarify the directionality between these factors by using more precise quantification of alcohol intake (e.g., daily alcohol intake in grams), controlling for metabolic baseline, or applying a longitudinal follow-up design.

A subgroup analysis was conducted on patients aged 18–25 years in the SG group. The results showed that the high-risk rate for AUDIT in this age group reached 42.86%, significantly higher than that in older patients; simultaneously, their BIS-11 impulsivity scores and VAS addiction behavior scores were also significantly elevated. This finding supports previous research suggesting that adolescents and young adults are more susceptible to addiction-related behaviors. Although the age variable did not emerge as an independent risk factor in the multivariate regression analysis, this may be because its effects were overshadowed by stronger predictive factors such as impulsivity and metabolic variables in the overall sample. Therefore, in preoperative assessment and postoperative intervention, the younger patient population (especially those aged 18–25) should be prioritized as a focus group. It is recommended to strengthen behavioral intervention and impulse control training for this population in postoperative psychological management.

The limitations of this study include its relatively small sample size and its single-center retrospective design, which introduces potential selection bias. Particular caution is warranted in interpreting the high-risk rate (42.86%) observed in the young subgroup (n=28) due to this small sample size. Therefore, this finding should be viewed as a preliminary hypothesis requiring validation in larger studies. To address these limitations and validate the current findings, future multicenter, prospective cohort studies are needed. Furthermore, building on existing knowledge that ghrelin hormone not only regulates appetite but also participates in alcohol reward behavior (29), future research could combine ghrelin level detection with neuroimaging techniques to further analyze its role in postoperative addictive behavior. Regarding our subgroup analysis, it focused exclusively on SG patients to elucidate surgery-specific pathways. Future studies with larger non-surgical cohorts are necessary to clarify whether similar metabolic-addiction relationships exist in obesity without surgical intervention.

## Conclusion

In summary, the elevated risk of postoperative AUD following SG is not only closely related to psych behavioral traits, such as increased impulsivity, but may also be influenced by glucose metabolism disorders. Therefore, glucose metabolism levels and psych behavioral characteristics should be key components of preoperative risk assessment. Based on the findings, it is recommended to establish a surgery-led multidisciplinary collaborative mechanism, involving referrals from surgery to endocrinology and psychology/psychiatry at 6, 12, and 24 months postoperatively; endocrinology would monitor glucose metabolism indices, such as FPG and HbA1c, every 3 months and dynamically adjust lifestyle interventions or medication regimens (including metformin) according to HOMA-IR; and psychological or psychiatric services would conduct quarterly assessments of impulsivity (BIS-11) and addictive behavior tendencies (VAS), implementing targeted cognitive behavioral therapy and positive thinking training. Simultaneously, the nutrition department would provide dietary support during periods of abstinence, thereby forming a closed loop of metabolic regulation and behavioral interventions for comprehensive postoperative management.

## Data Availability

The raw data supporting the conclusions of this article will be made available by the authors, without undue reservation.
